# Preclinical longitudinal imaging of tumor microvascular radiobiological response with functional optical coherence tomography

**DOI:** 10.1038/s41598-017-18635-w

**Published:** 2018-01-08

**Authors:** Valentin Demidov, Azusa Maeda, Mitsuro Sugita, Victoria Madge, Siddharth Sadanand, Costel Flueraru, I. Alex Vitkin

**Affiliations:** 1 University of Toronto, Department of Medical Biophysics, Toronto, Canada; 2 University Health Network, Princess Margaret Cancer Centre, Toronto, Canada; 3 Carleton University, Department of Systems and Computer Engineering, Ottawa, Canada; 4 University of Toronto, Department of Chemistry, Toronto, Canada; 5 National Research Council Canada, Information Communication Technology, Ottawa, Canada; 6 University of Toronto, Department of Radiation Oncology, Toronto, Canada

## Abstract

Radiation therapy (RT) is widely used for cancer treatment, alone or in combination with other therapies. Recent RT advances have revived interest in delivering higher dose in fewer fractions, which may invoke both cellular and microvascular damage mechanisms. Microvasculature may thus be a potentially sensitive functional biomarker of RT early response, especially for such emerging RT treatments. However it is difficult to measure directly and non-invasively, and its time course, dose dependencies, and overall importance in tumor control are unclear. We use functional optical coherence tomography for quantitative longitudinal *in vivo* imaging in preclinical models of human tumor xenografts subjected to 10, 20 and 30 Gy doses, furnishing a detailed assessment of vascular remodeling following RT. Immediate (minutes to tens of minutes) and early (days to weeks) RT responses of microvascular supply, as well as tumor volume and fluorescence intensity, were quantified and demonstrated robust and complex temporal dose-dependent behaviors. The findings were compared to theoretical models proposed in the literature.

## Introduction

Radiation therapy (RT), alone or in combination with other therapies, is one of the most commonly used treatment strategies for managing cancer. Typical clinical doses for targeting cancer cells in tumors are 2 Gy per fraction, administered daily for 5–6 weeks for a total cumulative dose of 50–70 Gy^1^. Such fractionation has been considered to be the most clinically effective, increasing the therapeutic ratio by repairing normal tissues and enhancing tumor cell kill compared to the equivalent single-fraction dose. However, recent advances in RT delivery and monitoring of the radiobiological tumor effects have led to the development of stereotactic body radiation therapy (SBRT), which delivers higher doses per fraction and fewer fractions for improved local control and lower damage to surrounding normal tissues^2^.

Preclinical studies provide emerging evidence that higher doses of radiation induce additional tumor cell kill through “non-classical” radiobiological mechanisms, mediated by tumor microvascular damage^3–6^. Specifically, Fuks and Kolesnick suggested that increased anti-tumor RT-effects are due to vascular damage^7–9^, with a minimum threshold dose of ~8–10 Gy^8^. Similarly, tumors receiving high dose RT were found to respond above the levels predicted with existing radiobiological models of cell death alone^10^. This was linked to the significantly increased proliferation rate of tumor vascular endothelial cells (EC) undergoing angiogenesis, potentially making tumor vasculature more sensitive to ionizing radiation^11^. Death of tumor EC was reported to initiate the inflammation cascade^12^, yielding hypoxic, acidic and nutrient deprived microenvironment, and enhancing radiation toxicity^13^.

In spite of intense research in this area, the underlying biological mechanisms of tumor response after high-dose RT remain unclear^14–17^. Little is also known about the dynamics of vascular changes, organization of tumor vasculature, angiogenesis and neovascularization at various post-RT stages. This is mostly due to the inability to study the dynamic response *in-situ* at the capillary level. A recent review of over 40 preclinical studies demonstrates lack of experimental consensus on the RT microvascular response^5^. The conflicting data arises from the variation in experimental protocols (animal models, cell lines, x-ray energies, dose levels), as well as differences in imaging and quantification techniques (immunohistochemistry *ex-vivo*, Doppler sonography and computed tomography *in-vivo*, etc.). Although some theoretical mechanistic models are proposed for RT vascular response effects, little direct experimental *in-vivo* data exists to support and validate these models. A good example is Kozin *et al*.’s model of neovascularization after high single-dose RT in rodents based on a thorough analysis of (conflicting) published data covering last 50 years of research in the field^18^. The numerous questions raised in this work about vascular dynamics in irradiated tumors demonstrate “… the urgent need for tracking vascular changes at the capillary level post-RT using advanced modern technologies” (ref.^18^). If successful, this line of research should enable better understanding of post-RT microvascular effects and provide early (inter-fraction) response metrics, potentially enabling personalization of the radiation treatments (adaptive RT). Addressing this problem is particularly timely because higher-dose radiation treatments such as SBRT, with their suggested greater involvement of the tumor microvasculature, are currently under active investigation in radiation oncology.

Tumor capillaries are known to be particularly sensitive to radiation^5^, but most of imaging modalities (ultrasound, magnetic resonance imaging, confocal fluorescence microscopy, etc.) do not have the requisite resolution capability or require potentially toxic contrast agents to visualize them and monitor their response longitudinally. Here we propose a new insight into the response of tumor microvasculature to RT using functional optical coherence tomography (OCT). OCT is an emerging label-free non-invasive 3D optical imaging modality for visualizing subsurface tissue details *in-vivo* at resolutions approaching microscopy and blood flow details at the microcirculation level^19^. Its functional extension called speckle variance OCT (svOCT) enables three-dimensional depth-resolved imaging of microvasculature *in-vivo* ^20^. The endogenous contrast of svOCT images originates from the different temporal light scattering properties between the blood within vessels and the surrounding “solid” tissues. Other than not requiring contrast agents, significant advantages of svOCT for tracking tumor vasculature post-RT include fast volumetric scanning (few seconds to a few minutes depending on the tumor size), rapid processing, 1 to 3 mm imaging depth (depending on tissue and tumor type), and blood flow/direction independence; this last characteristic is advantageous in that it maximizes microvascular detection and visualization, but may be a drawback if flow speed information is required. In addition, OCT scanners are now relatively cheap and portable.

The current “shedding light on radiotherapy” study builds on a decade of background work. Initially Mariampillai *et al*. developed svOCT method for microvasculature monitoring^21^. Leung *et al*. designed the heated animal restrainer for svOCT imaging, irradiation protocol and dose verification^22^. Maeda *et al*. optimized the well-established, but occasionally disadvantageous dorsal skin window chamber (DSWC) model^23^ and conducted a pilot study of a short-term response (2 weeks) to 30 Gy single-dose RT^24^. Conroy *et al*. developed post-processing techniques for vasculature quantification with biological metrics^25^. We build on this decade of previous experience, improving and refining essentially every aspect of this imaging and analysis platform, to now enable identification of vascular radiobiological response.

We selected the NOD-Rag1^null^ IL2rγ^null^ (NRG) mouse strain for this study because of its radio-resistant and immune-deficient nature^26^. Driven by current emerging clinical interest in SBRT for treating pancreatic cancer^27–29^, we used Bx-PC3 human pancreatic cancer cells to study its response to irradiation. From a variety of microvascular metrics developed by us and others over the years (vessel tortuosity, branching, length, fractal dimension, etc.^25,30–32^), here we report on the vascular volume density due to its calculation simplicity (number of vascular pixels divided by total pixels in the selected volume), robustness, minimal operator dependence and potential ease for results replication by other research groups. Two additional vasculature-independent measures were also performed for tracking RT response: tumor volume via caliper measurements and tumor cell fluorescence intensity via fluorescence microscopy after each svOCT imaging session. Tumor resections for histological staining and histopathologic evaluation were also performed at selected post-RT stages in several animals to support and validate the *in-vivo* longitudinal observations.

## Materials and Methods

### Mouse model, cell culture and tumor model

**Figure 1:**
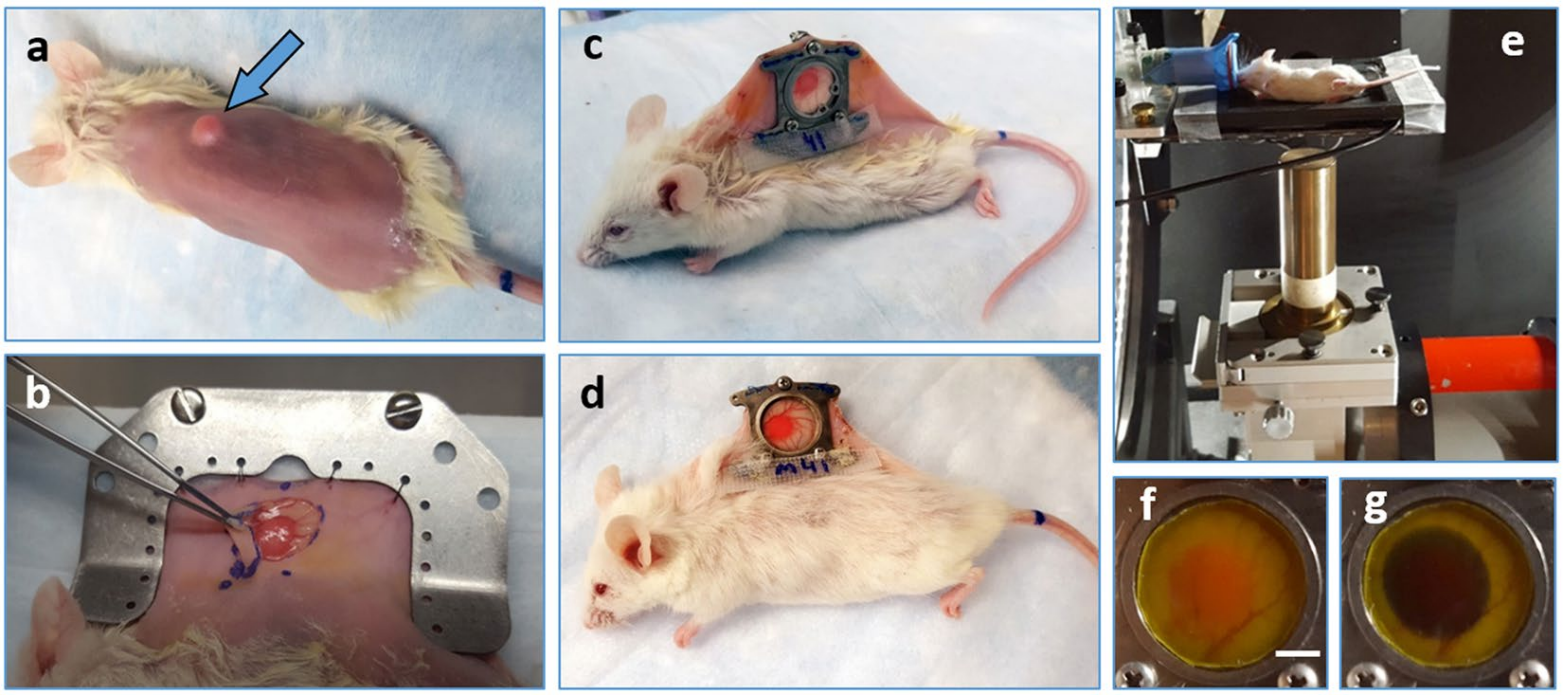
(**a**) NRG mouse with grown BX-PC3 tumor (blue arrow).The back was shaved prior to DSWC installation. (**b**) Flipped skin on the opposite side of the back showing the grown tumor during DSWC installation procedure. (**c**) Mouse with DSWC installed. (**d**) Same mouse as shown in (**c**) 9 weeks post-DSWC installation and 7.5 weeks post-RT (single-dose 20 Gy). (**e**) NRG mouse placed in the small-animal irradiator. 225kVp X-rays were incident from the bottom; white-light images showing (**f**) tumor location within a DSWC covered with EBT-2 Gafchromic film. (**g**) EBT-2 Gafchromic film color change after 10 Gy single-dose RT. Note that DSWC glass coverslip retaining ring was removed prior to RT to avoid possible dosimetric complications. Scale bar is 3 mm.

All animal procedures were performed in accordance with appropriate standards under protocol approved by the University Health Network Institutional Animal Care and Use Committee in Toronto, Canada (AUP #3256). Human DsRed-labeled BxPC-3 pancreatic cancer cells^33^ were purchased from AntiCancer Inc. (San Diego, CA, USA) and cultured in RPMI 1640 medium supplemented with 2 mM L-glutamine, 10% fetal bovine serum and 1% Penicillin Streptomycin (GIBCO BRL) at 5% CO_2_ and 37 °C. DsRed-labelled-BxPC-3 tumors were generated by injection of 2.5 × 10^5^ cells prepared in 10 μL of 1:1 PBS:Matrigel (BD Biosciences, ON, Canada) solution into the dorsal skin of seven- to eight-week-old NRG mice (Jackson Labs, ME, USA) using a 30 G needle. The DSWC surgery was performed 15–21 days post injection after the tumors reached 3–5 mm diameter (Fig. 1(a–c)). Titanium window chambers were surgically implanted into the dorsal skinfold of anesthetized (mixture of 80 mg/kg of ketamine and 5 mg/kg of xylazine) mice using the procedure described in ref.^23^. svOCT imaging was performed after a recovery period of three to five days post-DSWC installation. Optimized dorsal skin DSWC model allowed for monitoring the response for significantly longer period of time (Fig. 1(d)) compared to similar studies reported in the literature^34^.

### Tumor irradiation

Ionizing radiation was delivered to the tumor using a commercial small animal X-ray micro-irradiator system (XRad225Cx, Precision X-Ray Inc., North Branford, CT, USA) (Fig. 1(e)). With computer control, the system delivered single focal radiation beams (225 kVp, 13 mA, added filtration of 0.32 mm Cu) at doses of 10, 20 and 30 Gy with a diameter of 8 mm directly to BxPC-3 tumors, with a dose rate of 2.63 Gy/min. The X-ray tube was mounted on a rotating gantry with a flat panel detector located opposite the isocenter, which facilitated imaging and irradiation of the target at any given angle. The irradiator was calibrated to ensure accurate dose delivery with tissue phantoms using methods previously described^35^.

Prior to irradiation, mice were anesthetized using 5% isoflurane and maintained using 2% isoflurane delivered through a mask. In order to align the center of the tumor within the window chamber to the isocenter of the radiation beam, fluoroscopy images were taken and animal stage position adjusted accordingly. The location of RT and dose levels (Fig. 1(f) and (g)) were confirmed with calibrated Gafchromic EBT-2 film (ISP Inc., Wayne, NJ, USA) consisting of a radiosensitive monomer that polymerizes and changes color with absorbed dose.

### Experimental study schema

**Figure 2:**
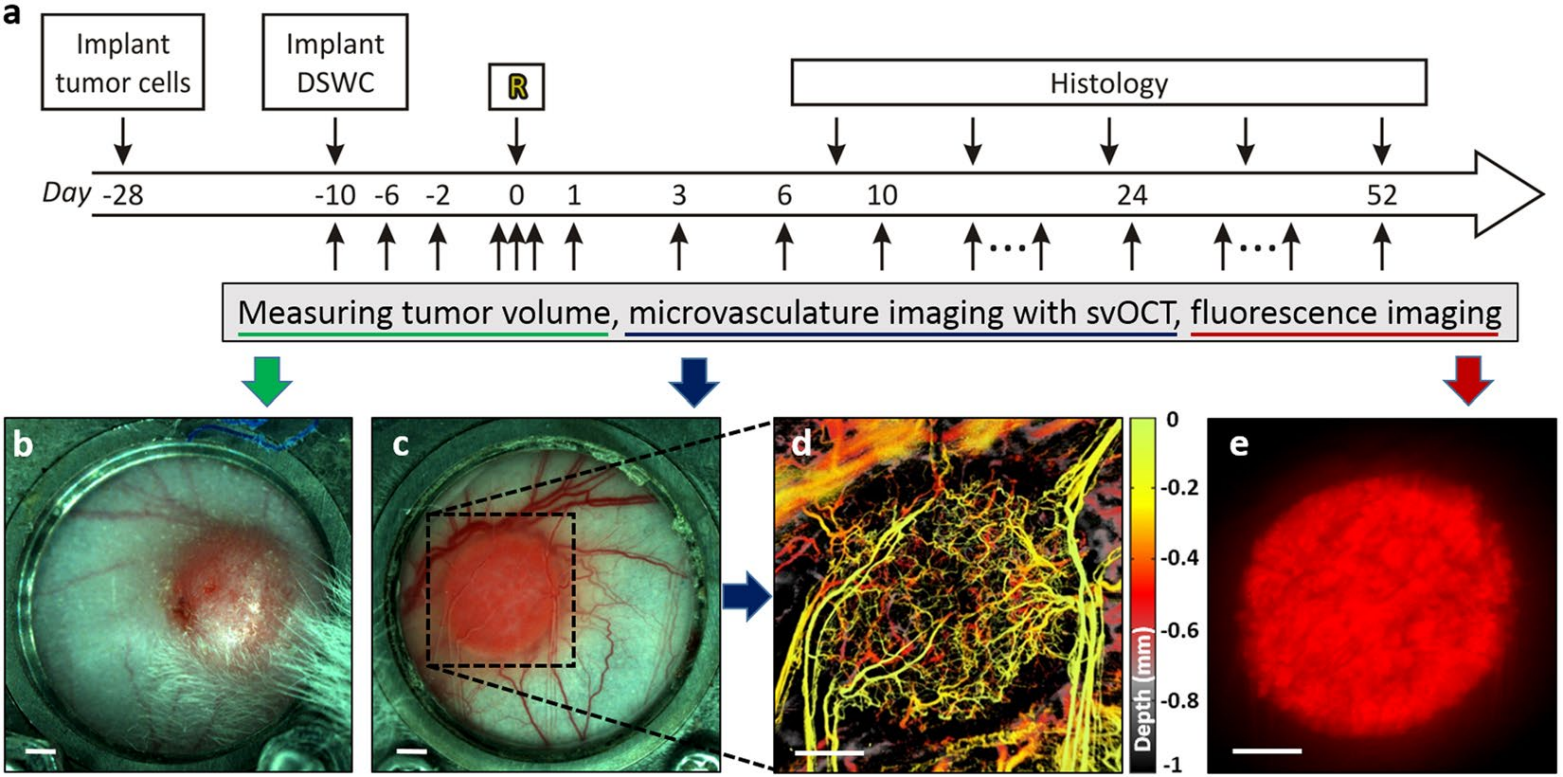
**(a)** Experimental time course. At day -28 tumor cells were injected into the dorsal skin. DSWC was implanted after the tumor volume reached 3–5 mm in diameter. Tumors were irradiated ~10 days after DSWC installation (day 0 labeled with “R”). Right after irradiation tumor vasculature was monitored within 90 minutes (minutes time scale). For five to eight weeks following irradiation, tumor changes were monitored repeatedly with caliper measurements (tumor volume), svOCT imaging (vasculature) and epi-fluorescence microscope imaging (cancer cell fluorescence). Tumor resection for histological staining was performed at selected post-RT stages in several animals to support and validate the *in-vivo* observations. White light images of the **(b)** back and **(c)** front of the tumor within window chamber. **(d)** svOCT microvasculature map of the area labeled with black dotted rectangle in **(c)**. **(e)** Tumor cells Ds-Red fluorescence image. Scale bars are 500 μm.

The time course of conducted experiments is shown in Fig. 2(a). Initially, tumor cells were injected into the dorsal skin. After the tumor volume reached 3–5 mm in diameter ~2 weeks later, the DSWC was implanted. Irradiation was performed ~10 days after DSWC installation. This delay ensured adequate tumor and vascular growth, assessed by periodic svOCT and fluorescence imaging. At day “R”, tumor was treated with a single-dose of radiation using the small animal irradiator. For five to eight weeks following irradiation, tumor changes were monitored repeatedly with caliper measurements (tumor volume), svOCT imaging (vasculature), and epi-fluorescence microscopy (tumor cell status). Specifically, tumor size at the back side of the window chamber (Fig. 2(b)) was measured in three perpendicular directions with calipers prior to every imaging session. svOCT from the front side of the window chamber (Fig. 2(c)) was used to image tumor microvasculature (Fig. 2(d)) within the area labeled by the black rectangle. DsRed (535 nm excitation, 580 nm emission) tumor cell fluorescence images (Fig. 2(e)) were obtained with an epi-fluorescence microscope with consistent exposure settings (Leica MZ FLIII, Leica Microsystems, Richmond Hill, ON, Canada), and analyzed using MATLAB by computing the average intensity of all pixels.

To support longitudinal *in-vivo* observations, several animal were sacrificed and tissue sections were histologically stained at various time points. Mice were euthanized by anesthesia with ketamine/xylazine followed by cervical dislocation. Tumors were resected, fixed in 10% formalin and processed for histologic staining. Hematoxylin and eosin (H&E) were used to view cellular morphology, and labeling of DNA fragments (TUNEL antibody assay) was used to quantify cellular apoptosis. Slides were scanned by Aperio Scanner, and TUNEL positivity was measured for the entire tumor section using Aperio ImageScope software (Leica Biosystems, Concord, ON, Canada).

### Optical coherence tomography system

**Figure 3:**
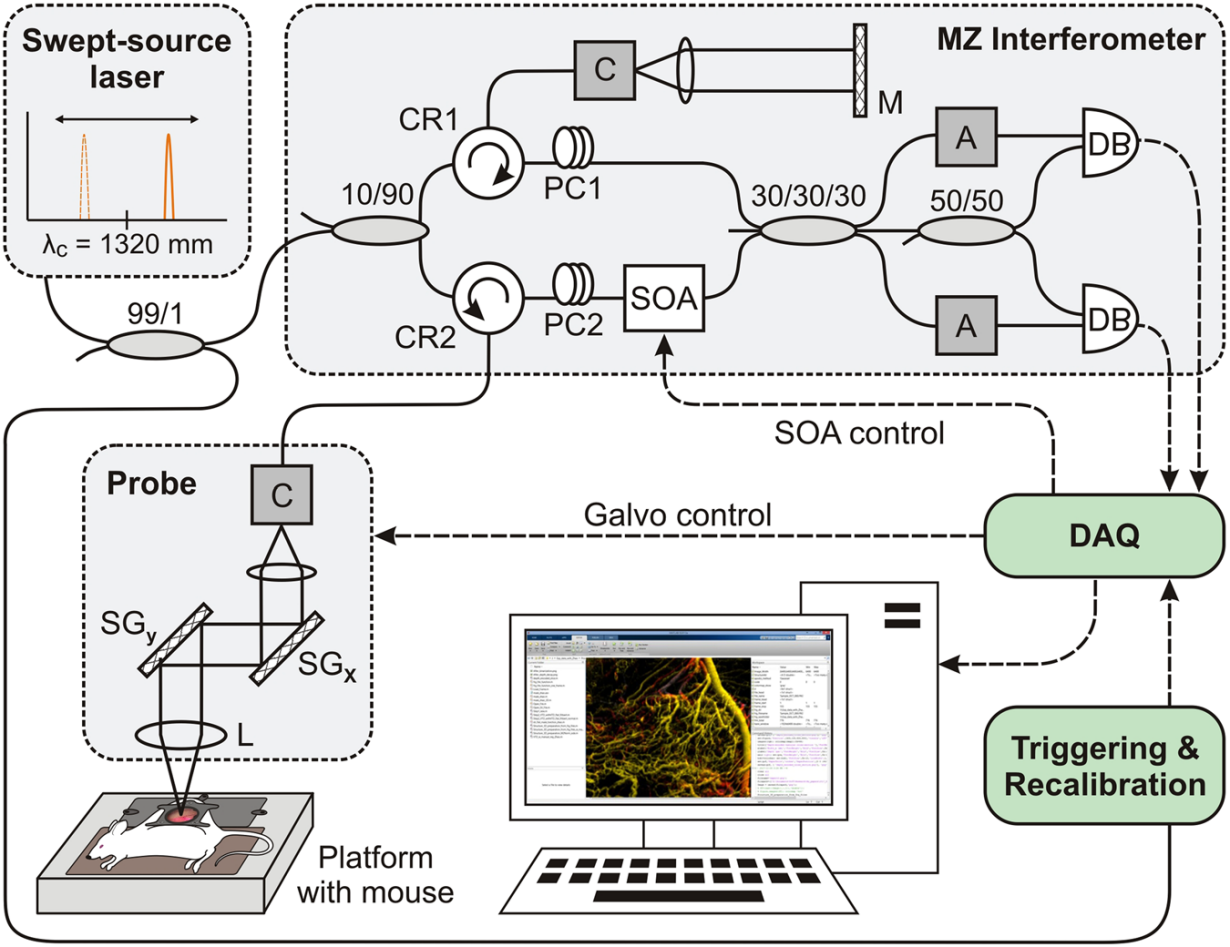
Schematic diagram of the swept-source OCT system setup with quadrature Mach-Zehnder fiber-based interferometer and optical amplification: SOA - semiconductor optical amplifier, PC - polarization controller, A - fiber attenuator, DB - dual balanced photo-detector, DAQ - data acquisition card, C - collimator, MZ Interferometer - Mach-Zehnder interferometer, L - lens, M - mirror, SG - scanning galvo, CR - circulator.

All OCT images were acquired using a previously-described swept source OCT system based on a quadrature interferometer to suppress the complex conjugate artifact, as shown in Fig. 3 (refs^36,37^). Briefly, the source (HS2000-HL, Santec, Japan) had a central wavelength of 1320 nm, a full width at half-maximum wavelength of 110 nm and an average output power of 10 mW. The repetition scan rate of the source was 20 kHz with a duty cycle of 68%. The light output was split in the first 2 × 2 coupler and 90% was directed toward the tissue. A 2D galvo scanning system enabled lateral beam translation and thus 3D volumetric imaging (GVS-012, Thorlabs, NJ, USA). Tissue back-scattered light was coupled back within the optical fiber and fed into a semiconductor optical amplifier (SOA - BOA1017, Covega, MD, USA), with gain adjusted to 35 dB, to boost the signal level. The SOA had the same center wavelength and bandwidth as the laser source. We used a polarization controller (located before the SOA) to minimize the differences between the shape of normalized light spectra in the reference arm and after the SOA. The amplified signal was combined with the reference signal in a 3 × 3 coupler followed by a 2 × 2 coupler. Two channels balanced detection was used to extract the complementary components of the complex interferometric signal. Two attenuators were used to match the optical power entering the balanced detectors (PDB150C, Thorlabs, NJ, USA) with a saturation level of 5 mW. Two detector outputs were digitized using a data acquisition card (ATS9625, Alazartech, Montreal, Canada) with 16-bit resolution and sampling rate of 250MS/s. The resultant axial and lateral resolutions (in air) were 8 µm and 15 µm, respectively. To ensure consistency of obtained *in-vivo* data over time and between animals, OCT optical power at the probe output was measured before each imaging session to be 5 mW, OCT probe imaging angle was set to 84°, relative to horizontal, and imaging speed was fixed at 40 frames per second.

### Imaging, data processing and representation

#### svOCT

**Figure 4:**
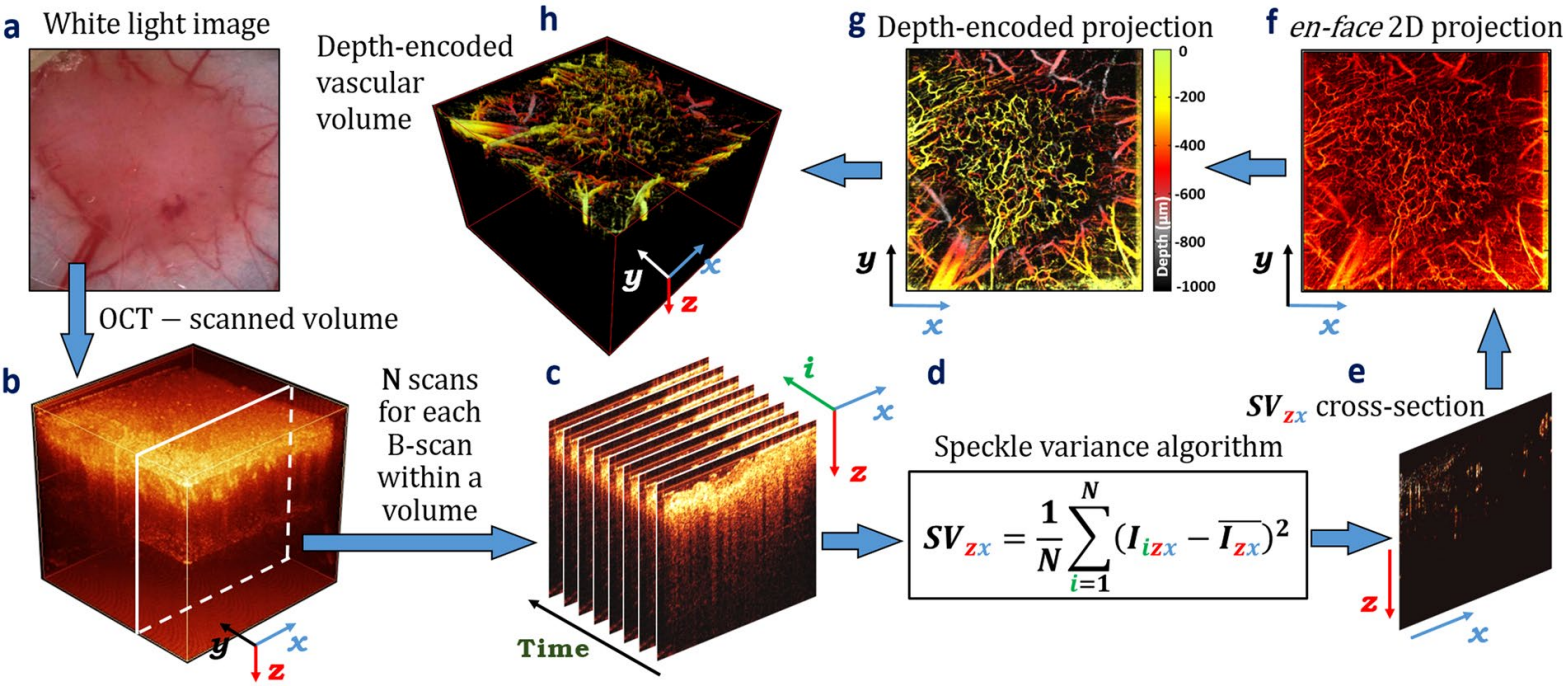
Real-time imaging of tumor microvasculature via svOCT. **(a)** White light image of window chamber with tumor tissue under the glass coverslip. **(b)** OCT volumetric image of (**a**) taken over a 6 × 6 mm^2^ field of view. **(c)** 8 sequential same-location B-scans for further inter-frame comparison required for svOCT analysis. **(d)** The svOCT algorithm for inter-frame comparison of pixel texture. **(e)** ${{\boldsymbol{SV}}}_{{\boldsymbol{zx}}}$ vascular cross-section obtained with svOCT algorithm. **(f)** Vascular *en-face* two-dimensional projection. **(g)** Depth-encoded vascular en-face two-dimensional projection. **(h)** Depth-encoded vascular volume of tissue volume shown in (**b**).

Tumor-bearing mice (n = 60 with 45 irradiated and 15 non-irradiated tumors) were anesthetized by inhalation of 2% isoflurane and placed on a mouse restrainer^22^ with built-in 37 °C heating element to prevent motion artifacts and maintain physiological temperature during imaging procedures. OCT volumetric images (Fig. 4(b)) were taken over a 6 × 6 mm^2^ field of view with 800 A-scans per frame and a gate length of N = 8 (number of sequential same-location B-scans), to enable inter-frame comparison required for svOCT analysis (Fig. 4(c)). This gate length may be optimal for low bulk tissue motion scenarios, such as the DSWC^20^. The svOCT algorithm (Fig. 4(d)) was used to calculate the inter-frame intensity variance from the same spatial location, with the contrast arising from differences in time-varying speckle properties at each pixel:

1 $$\,S{V}_{zx}=\frac{1}{N}\sum _{i=1}^{N}{({I}_{izx}-\overline{{I}_{zx}})}^{2}$$

where N is the number of B-scans acquired at the same spatial location within a tissue volume, ${I}_{{izx}}$ is the intensity of the (*z,x*)^th^ pixel of the *i*-th B-scan, *z* is the axial coordinate, *x* is the lateral coordinate and $\bar{{I}_{{zx}}}$ is the mean intensity of *i* pixels from N consecutive B-scans. This procedure was then repeated for all spatial locations within the scanned tissue volume to obtain ${{SV}}_{{zx}}$ vascular cross-sections, as shown in Fig. 4(e).

In Eq. (1), if *N* B-scans are acquired faster that the “stationary” solid-tissue decorrelation time, then the value of (${I}_{{izx}}-\bar{{I}_{{zx}}}$) for these pixels approaches zero, thereby suppressing the tissue signal in the resulting SV image. Here, the B-scan acquisition rate was set to 25 ms: this was fast enough that signals from stationary tissues did not de-correlate between frames (thus ~0 svOCT_signal_), while being sufficiently slow to ensure complete inter-frame de-correlation for pixels representing vascular blood (thus high svOCT_signal_).

Volumetric vascular images were composed of hundreds/mm of ${{SV}}_{{zx}}$ vascular cross-sections taken in lateral $y$ dimension. Those images were post-processed for vascular volume density (VVD) calculation, vascular *en-face* 2D projection (Fig. 4(f)) and depth encoded 2D (Fig. 4(g)) and 3D (Fig. 4(h)) representation using (i) morphological opening/closing algorithm for noise and artifact removal^38^ to minimize contributions from non-vessel signals such as bulk tissue motion; (ii) binarization with Otsu’s thresholding method^39^ in the depth direction to retain deep-vessel information otherwise suppressed due to the exponential attenuation of the OCT signal; (iii) tumor surface masking and leveling for correct depth encoding while preserving blood vessel topology, orientation and connectivity. VVD was calculated as a fraction of vascular pixels of the total number of pixels in the analyzed volume. Green-yellow-red-grey-black color map (256 color gradations) was chosen for depth-encoding (green = top tissue layers just below the glass coverslip, black = deepest tissues). Matlab software (Mathworks, MA, USA) was used for processing the data.

### Scientific rigour and statistical considerations

Many literature studies of radiobiological microvascular responses often report conflicting results, likely due to the variations in experimental protocols (animal and tumor models, irradiation methods, etc.), imaging methodologies, and quantification techniques^5^; these difficulties underscore the subtle and complex nature of the problem. After more than a decade of careful background preparation, the current study finally ensures robust and unbiased experimental design and analysis of results, rigorously quantifying vascular radiobiological response of irradiated tissues. For the three reported dose levels of 10, 20 and 30 Gy, a total of 60 mice were used: 15 animals for each dose plus 15 un-irradiated. As some animals were used for validating histology, animal numbers reduced towards the latest time points (~8 weeks), from 15 to 7–8. This relative reduction of animal numbers is reflected in the size of error bars in the plots reported below; the initially large number of 15 animals per dose was chosen to ensure robust results throughout the imaging time course regardless of this histological attrition. Further, as sex is an important and potentially confounding biological variable, only female mice were used to exclude this uncertainty. A new batch of pancreatic tumor cells and ‘fresh’ chemicals were purchased from official vendors to further reduce the risk of laboratory-to-laboratory differences and increase rigor and robustness of the reported trends.

Repeated measures analysis of variance (ANOVA) was performed using SPSS Statistics software (IBM, Armonk, NY). Two-way repeated measures ANOVA with Bonferroni post-test was used for serial imaging data to compare the results for the groups irradiated with different doses. The number of samples (n) indicates the number of mice per treatment group. In all cases, *P* < 0.05 was considered statistically significant, and all error bars represent mean ± standard deviation.

## Results and Discussion

**Figure 5:**
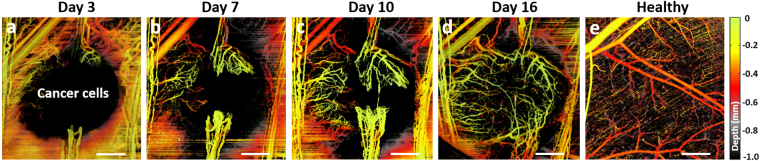
BX-PC3 pancreatic tumor vasculature development within 16 days after subcutaneous injection of cancer cells into dorsal skin. **(a)** 3 days; **(b)** 7 days; **(c)** 10 days; **(d)** 16 days after injection. **(e)** Healthy tissue vasculature for comparison. Scale bars are 0.5 mm.

svOCT imaging and its associated post-processing steps provide a powerful platform for assessing volumetric tumor vasculature growth and response to radiation. As seen in Fig. 5, pancreatic tumor xenograft vasculature aggressively developed within ~2 weeks after subcutaneous injection of tumor cells into dorsal skin. At day 3 after injection seen in Fig. 5(a), there was microvascular growth from neighboring normal tissue vessels^40^. Vessel growth continued, forming the “claws” as seen at one-week time point in Fig. 5(b). After these connect at ~day 10 (Fig. 5(c)), further new vessels quickly sprout inside the tumor to fully vascularize it within a few days (day 16 in Fig. 5(d)). The structure of vascular bed in tumor is seen to be markedly different from that in normal tissue, where microarchitecture of vascular network is more hierarchically organized as shown in Fig. 5(e), with more ordered and evenly distributed vessels to allow adequate perfusion of nutrients and oxygen to all cells^41^. In contrast, tumor vessels are immature, tortuous, irregular in diameter, and often sharply bent. They form a disorganized labyrinth with a lack of conventional blood vessel hierarchy in which arterioles, capillaries, and venules are not clearly identifiable^42^.

**Figure 6:**
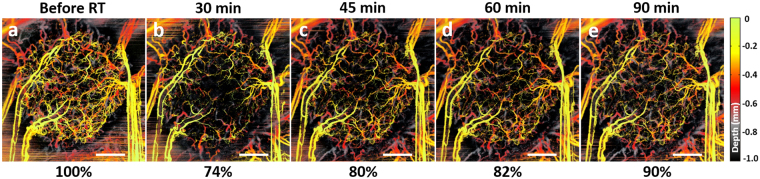
Immediate (minutes - 10s of minutes) tumor microvascular response within 1.5 hours post-RT 10 Gy single-dose. **(a)** Before irradiation; **(b)** 30 min after; **(c)** 45 min after; **(d)** 60 min after; **(e)** 90 min after irradiation. Scale bars are 1 mm. The numbers below each panel represent VVD, relative to the pre-radiation value.

Introducing single-dose radiation treatment into this course of tumor development changes its growth dynamics. Prior to examining longer-term responses (days-weeks), we look closely at the immediate (minutes-scale) response. In other tumor types^43,44^ and in our earlier investigations using intravital microscopy^45^, irradiation with high single doses causes rapid vascular alterations in human tumor xenografts. Depth-encoded svOCT panels in Fig. 6 demonstrate the immediate microvascular effects following 10 Gy irradiation. The vascular volume density (VVD) markedly decreased by 26% half an hour post-RT (Fig. 6(b)) from its initial state before irradiation (Fig. 6(a)). Interestingly, maximum response at this time point is seen in small vessels (10–30 μm in diameter); small-to-medium size vessels (30–70 μm in diameter) appear less affected.

Many of these alterations seem non-permanent, with majority of these vessels re-appearing later: at 45 min and 60 min time points, the circulation recovery was detected (Fig. 6(c,d)) reaching 90% of initial vascularity at 90 min post-RT (Fig. 6(e)). This may be an indication of temporary transient microvascular thrombosis or capillary anastomosis bypass after irradiation^24,45,46^. In other words, those vessels that reappeared at later time points post-RT were not permanently damaged by irradiation. Permanent disappearance of ~10% of vessels may be an indication of radiation-induced death of endothelial cells and collapse of the fragile tumor vessels as a result of an interstitial fluid pressure elevation caused by extravasation of plasma proteins^47,48^.

**Figure 7:**
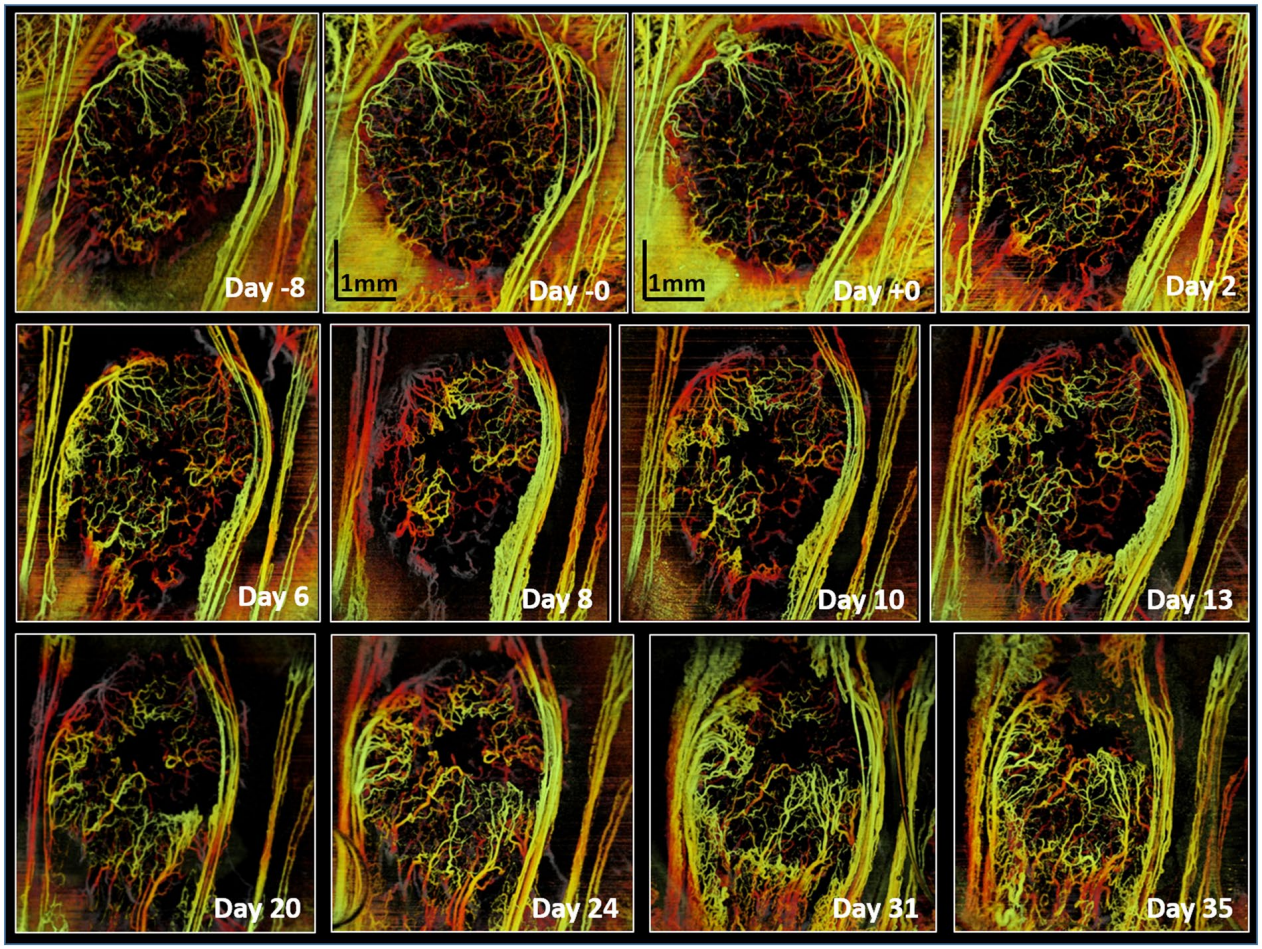
Longitudinal OCT imaging of radiation response of the tumor vasculature to single dose of 20 Gy. svOCT images were taken before and multiple times following irradiation. “Day −0” image was taken 1 hour pre-RT and “Day +0” represents 1.5 hours post-RT. Maximum suppression of tumor vasculature is ~ at day 8, followed by vascular re-growth from the vessels outside the tumor. Scale bars = 1 mm.

Figure 7 shows the effect of a 20 Gy single dose on tumor microvasculature over 6 weeks (from 1 week pre-RT to 5 weeks post-RT). The tumor was irradiated after being fully vascularized (“Day -0”). Initial response is seen at 1.5 hours after irradiation (“Day +0” image), where VVD = 83% of pre-RT vasculature; svOCT images at t = 2, 6 and 8 days post-RT clearly demonstrate that vessels in the tumor core are preferentially affected compared with those in the tumor rim. This supports the previous conjecture that parts of vascular networks in the tumor periphery are ~ normal tissue blood vessels sprouting by angiogenesis into the tumor mass^18^; these might be more resistant to radiation compared to the new tumor blood vessels in the core formed by vasculogenesis^49,50^.

Data beyond ~10 days provides clear evidence of tumor re-vascularization via growth of the surviving vessels, in accord with earlier studies and hypothesis that tumor regrowth after local irradiation is dependent on blood vessel formation by surviving endothelial cells^18,51^. It is also interesting to note that throughout this > 10 days revascularization process, the tumor region appears to be getting smaller.

**Figure 8:**
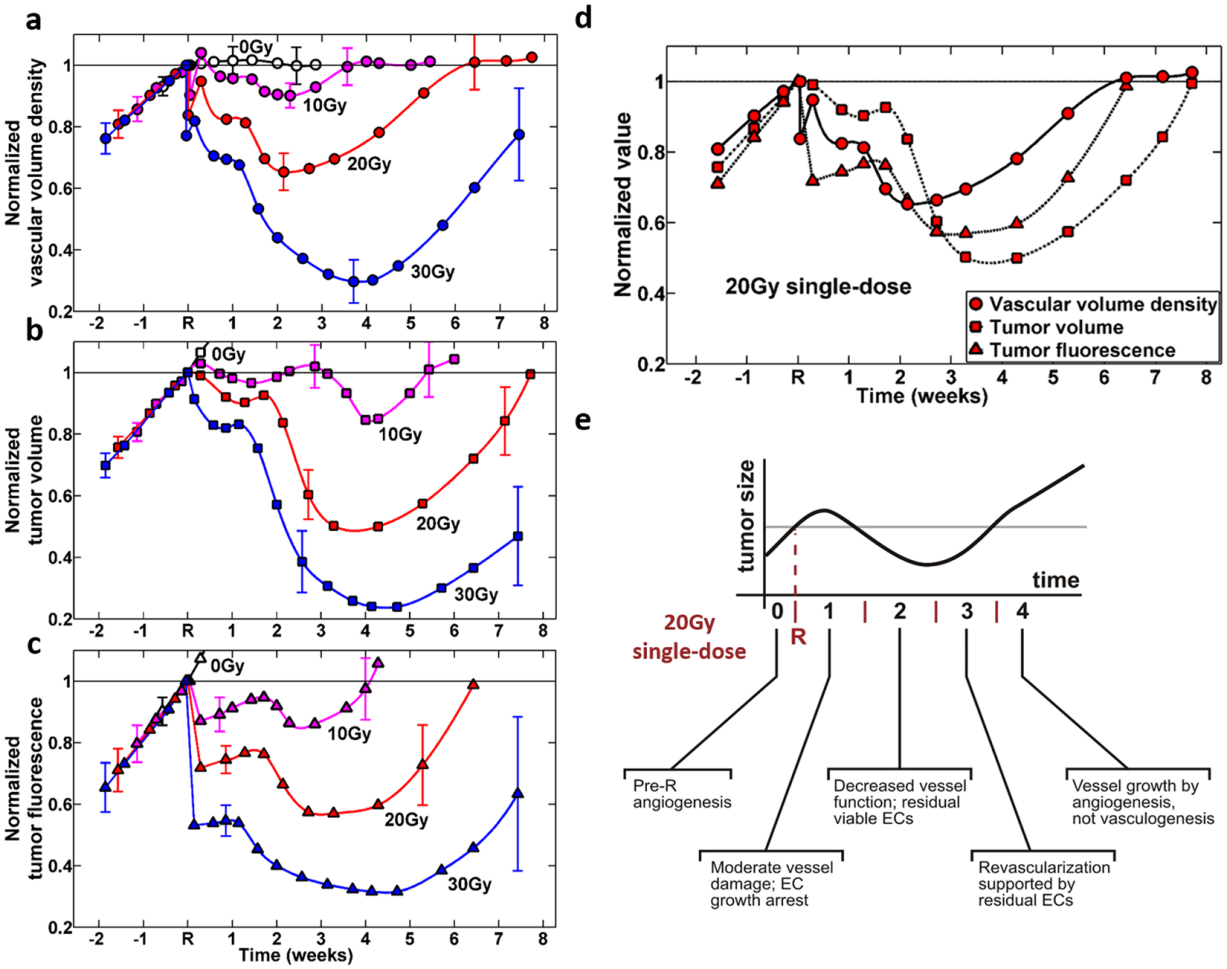
Tumor development dynamics as reflected via changes in vascular volume density (VVD), tumor volume and fluorescence intensity after 10, 20 and 30 Gy single-dose local irradiation. **(a)** Tumor microvasculature response. **(b)** Tumor volume response. **(c)** Tumor fluorescence response. Quantified values of VVD, volume and fluorescence pre- and post-RT changes of each tumor were normalized to values of these metrics obtained 1 hour before irradiation of the same tumor. Non-irradiated tumors (0 Gy data) continued to grow and were sacrificed at earlier time points for humane reasons (tumors grew too big and data is not shown for the entire course of 7.5 weeks post-RT). (**d**) Summary graph for VVD, volume and fluorescence response to 20 Gy single-dose irradiation. In (**a**)–(**d**), symbols are experimental points, and lines are a guide for the eye. n = 7–15 per group, error bars = mean ± standard deviation. (**e**) A recently proposed literature model of tumor growth and corresponding changes in microvasculature following 20 Gy single-dose irradiation (after ref.^18^).

The individual ‘case studies’ presented above are interesting and do provide some insights, but the real value of the developed svOCT platform is in its large imaging throughput capability and quantifiable metric extraction. We thus present a quantitative summary of the entire n = 60 animal study, for the three irradiation dose levels (plus un-irradiated controls) showing the three measured variables: tumor VVDs extracted from svOCT images (Fig. 8(a)), volumes from caliper measurements (Fig. 8(b)), and fluorescence intensity from microscopy (Fig. 8(c)).

The longitudinal monitoring data is shown over the entire ~10 week temporal observation interval. Error bars are calculated at each experimental point, but are only shown at selective intervals for clarity – in the pre-RT regime, in the midcourse, and towards the end of the post-RT observation interval. Figure 8(d) shows the three metrics on a single panel for the 20 Gy dose case. Also shown in Fig. 8(e) is the proposed literature model^18^ for the temporal course of microvascular changes post RT; as mentioned previously, this model was not based on direct experimental observations. It will be used here to help interpret the derived experimental data of Fig. 8(a)–(d).

Starting with Fig. 8(a), several important trends of tumor microvascular response to single-dose irradiation become evident:

- irradiation inhibits VVD (for up to 2–4 weeks following irradiation);
- magnitude of inhibition increases with dose levels (~10% drop 2 weeks after 10 Gy, ~70% drop 4 weeks after 30 Gy); the decrease is temporary (thus single dose is not enough to permanently control the tumor), and eventually VVD returns to pre-irradiation levels;
- time-to-return increases with dose (~3.5 weeks for 10 Gy, > 8 weeks for 30 Gy);
- tumor microvasculature response within 1.5 hours after irradiation is more pronounced for higher doses (23% of microvessels exhibited temporary shutdown after 30 Gy, versus 10% after 10 Gy). The majority of these were seen to be microvessels of less than 30μm in diameter;
- the described statistical analysis of variance was performed on the three irradiated and one un-irradiated control group over the course of corresponding temporal trajectories, to check if the four dose cohorts were indeed different from each other. For t > 1.5 weeks, this was definitely so, with P-values in the 0.0001–0.01 range. Immediately following irradiation for up to 1–1.5 weeks, the situation was ambiguous, with P-values in the 0.03–0.15 range (largest P-value for the 10Gy-to-0Gy cohort difference at t < 1 weeks). We thus conclude that the differences in the temporal trajectory of the microvascular response increase with dose, and take ~1–1.5 weeks to manifest unequivocally.

These direct and robust experimental observations of longitudinal microvascular RT response *in-vivo* yield solid results for *de-novo* mechanistic model development, and can also serve as empirical foundation/validation for previously-proposed models (e.g., one shown in Fig. 8(e), as discussed below).

Tumor volume response to different doses (Fig. 8(b)) also demonstrates complex dynamics over the monitored time period, its overall shape and dose dependence being somewhat similar to the VVD behavior. The temporal response is overall slower than the microvasculature, in that the maximal tumor shrinkage (minimum tumor volumes) are reached at t ~ 4–5 weeks following dose deposition, independent of dose levels. This sequence of radiation damage events – first microvascular response followed by cellular/tissue shrinkage – makes sense in light of existing radiobiological models mentioned previously^5,18^. It also suggests that functional imaging approaches, such as svOCT that target earlier-responding microvasculature may indeed be preferable for potential treatment adjustment/personalization compared to ‘conventional’ anatomical tumor-volume-based imaging methods (e.g., x-ray based portal imaging or cone-beam CT^52^). Analogous to VVD, we note that the maximal tumor shrinkage increases with dose (10 Gy – 20%, 30 Gy – 80%), and the time to initial volume recovery is also dose dependent (10 Gy – 5.5 weeks, 20 Gy – 8 weeks, 30 Gy > 8 weeks (beyond our experimental observation interval). There is also some indication of complex early growth inhibition (without significant shrinkage) for the first 1.5–3 weeks following irradiation followed by a rapidly accelerating rate of tumor volume decrease (nadir at 4–5 weeks), and then recovery. These experimental observations will (1) need to be examined in additional tumor models to test and verify their generalizability and (2) will have to be accounted for in future predictive radiobiological models that can explain such complicated growth dynamics, including the complex interplay between vascular and cellular compartments.

Tumor DsRed fluorescence intensity has been reported to indicate cancer cell viability levels^53^ and to serve as indirect measure of the proportion of hypoxic cells in the tumor^45^. Figure 8(c) shows the response curves of this metric for the three doses. Once again, the general shape of the curves is similar to that of VVD and tumor volume, with greater resemblance to the latter; one significant difference is the sharp drop in fluorescence intensity very early following irradiation. Specifically, within one day post-RT, a significant decrease is seen in tumor cell fluorescence intensity (14% drop for 10 Gy, 30% for 20 Gy, and 48% for 30 Gy). There follows a 1–2 week long slight increase, followed by another drop (nadirs at 3 weeks and 85% for 10 Gy; 4 weeks and 60% for 20 Gy; and 5 weeks and 30% for 30 Gy). The subsequent time-to-recovery is also dose-dependent – 4 weeks (10 Gy), 6.5 weeks (20 Gy) and >7.5 + weeks (30 Gy).

To help better understand and interpret the various inter-connected temporal behaviors of the three measured metrics, we present all three on the same panel for a single dose of 20 Gy (Fig. 8(d)). Shortly after irradiation, tumor cell fluorescence intensity markedly drops together with collapse of tumor capillaries and small vessels followed by vasculature partial recovery within first two days. Tumor cell kill (a combination of direct cellular damage and vascular induced cell death mechanisms) causes tumor growth arrest with gradual tumor size decrease within first 10 days. For the latter mechanism, it was pointed out in 1980s that one endothelial cell subtends a segment of a tumor containing as many as 2,000 tumor cells^54^; thus collapse of one endothelial element of the down-stream blood flow may cause an avalanche of tumor cell death along the defunct vessels (as seen, the VVD response is ahead in time and in magnitude, compared to tumor volume). As the tumor shrinks and the VVD metric exhibits a plateau, more cancer cells gain access to oxygen and nutrients which may be the reason of temporary tumor relapse (t~2 weeks). Despite this brief increasing volume trend, number of blood vessels continues to decrease, likely causing localized hypoxia (tumor fluorescence curve), and leading to significant tumor shrinkage down to 45% of original size at week 4 post-RT. Tumor re-vascularization by growth of the surviving vessels starts after ~2 weeks post-RT. It is noted that as vessels start to re-grow and sprout into the tumor from surrounding tissues (Fig. 6, days 13–35), tumor fluorescence intensity starts to grow as well (with a few days delay). Several days after that, the tumor shrinkage stops, following by the complete regrowth at 7.5 weeks. Teasing out the passenger versus driver effects – in other words, which of the recorded metrics is/are the primary cause(s) and which is/are the resultant effect(s) in the observed tumor response – is not directly evident from the data and was not meant to be addressed in this study. Indeed, additional radiobiological experiments and correspondingly advanced radiobiological models may be needed. For now, this robust and previously unavailable experimental data can serve as an important foundation for hypothesis-generating research.

Such advanced radiobiological models are indeed starting to appear in the literature. An example is shown in Fig. 8(e), put forth by Kozin *et al*.^18^ in 2012, based on the varying (and often conflicting) reports of irradiated tissue studies to date. Despite sub-optimal data for hypothesis generation, these authors were able to propose purported mechanisms of microvascular dynamics following high single dose of radiation, including the resultant effects of tumor volume shrinkage and subsequent regrowth. As seen in Fig. 8(e), the general shape of the theoretically predicted tumor volume curve (with purported vascular mechanisms shown along the abscissa axis) agrees well with the experimental data of our study (particularly VVD and tumor volume metrics of Fig. 8(a) and (b), respectively). In this context, these results can be seen as the direct and successful response to Kozin *et al*.’s charge to “… the urgent need for tracking vascular changes at the capillary level post-RT using advanced modern technologies” (ref.^18^). It will be interesting to see how this and related radiobiological models will be adjusted in light of the detailed results presented in this paper.

The exploration of additional microvascular metrics may provide more insights into radiation-induced tumor vascular response and, importantly, prediction of therapeutic outcomes. Among those in OCT angiography research, most promising may be vessel tortuosity (to evaluate the efficiency of blood transport and vascular remodeling), total and average vessel lengths (to measure vessel/capillary pruning), fractal dimension (to quantify the vascular space-filling properties and vascular network complexity), and tissue vascularity (to identify tumor regions that are likely to be hypoxic)^25,30–32^.

**Figure 9:**
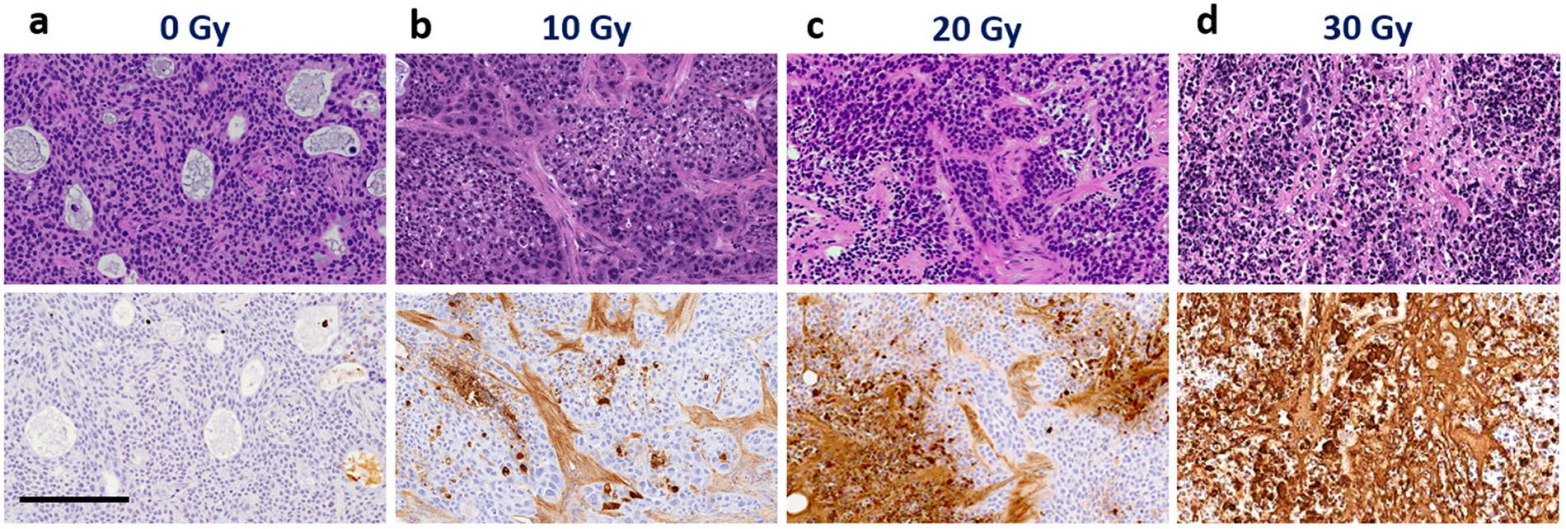
Representative images of H&E (top row) and TUNEL (bottom row) staining for tumors irradiated with **(a)** 0 Gy; **(b)** 10 Gy; **(c)** 20 Gy; **(d)** 30 Gy. Tumors were resected 2 weeks following irradiation. TUNEL positivity for the whole tumor section was 0.3%, 15.8%, 47.6%, and 97.7% for 0, 10, 20, and 30 Gy, respectively. Scale bar = 200 μm.

Fig. 9 presents Hematoxylin and Eosin (H&E) and TUNEL staining of tumor regions for the control and the three irradiated (10, 20 and 30 Gy) cohorts. Representative images at selected times (t = 2 weeks here) following irradiation are shown. Control staining (Fig. 9(a)) shows that tumor cells are in active proliferation state prior to irradiation (TUNEL), with many small and medium vessels (H&E). Two weeks following 10 Gy (Fig. 9(b)), mainly stromal cells are affected, with 16% of cancer cells undergoing apoptosis (TUNEL). Moderate damage (48%) is seen at 2 weeks post 20 Gy (Fig. 9(c)) to cancer and stromal cells (TUNEL). Finally Fig. 9(d) shows almost complete damage (98%) of stromal and cancer cells in tumor core (TUNEL) at 2 weeks post 30 Gy RT. Similar to the tumor *in-vivo* dynamics, these histological *ex-vivo* discrete point snapshots underscore the importance of (1) dose level and (2) time post-RT in furnishing the complex trajectory of tumor radiobiological response.

## Conclusion

This study presents comprehensive experimental results from *in-vivo* pancreatic human tumor xenografts subjected to three different radiation dose levels, with noninvasive imaging performed for up to 8 weeks following dose deposition. A novel state-of-the-art functional OCT microvascular imaging and quantification platform was developed, refined and validated specifically for this radiobiological monitoring study. Additional *in-vivo* measures, including tumor size and fluorescence, were also collected to supplement the obtained microvascular information. Complicated temporal trajectories of radiation response metrics were found, and compared with emerging radiobiological models of microvascular radiation response. The reported vascular volume density metric was simple to calculate, proved robust and was suitable to reflect the first-order changes in tumor microvasculature response to radiation. Higher-order metrics such as fractal dimension, average Euclidean vessel segment length, total length of smallest detectable vessels (~capillaries) and others^25,32^ that require additional signal processing/image analysis steps, may offer further useful quantifications. Study of their suitability to radiation-induced vascular changes is currently ongoing, as are our initial attempts to apply radiomics/machine learning approaches^55,56^ to the entire measured longitudinal RT response parameter space. Future work will examine the generalizability of these results in other preclinical *in-vivo* models, explore the effects of fractionation (multiple dose treatments), and further expand our OCT-based “shedding light on radiotherapy” platform into pilot clinical studies of microvascular radiotherapeutic monitoring of patients^57,58^.
